# Bone Balance within a Cortical BMU: Local Controls of Bone Resorption and Formation

**DOI:** 10.1371/journal.pone.0040268

**Published:** 2012-07-23

**Authors:** David W. Smith, Bruce S. Gardiner, Colin Dunstan

**Affiliations:** 1 School of Computer Science and Software Engineering, The University of Western Australia, Crawley, Western Australia, Australia; 2 School of Aeronautical, Mechanical and Mechatronic Engineering, The University of Sydney, Sydney, New South Wales, Australia; The Hebrew University, Israel

## Abstract

Maintaining bone volume during bone turnover by a BMU is known as bone balance. Balance is required to maintain structural integrity of the bone and is often dysregulated in disease. Consequently, understanding how a BMU controls bone balance is of considerable interest. This paper develops a methodology for identifying potential balance controls within a single cortical BMU. The theoretical framework developed offers the possibility of a directed search for biological processes compatible with the constraints of balance control. We first derive general control constraint equations and then introduce constitutive equations to identify potential control processes that link key variables that describe the state of the BMU. The paper describes specific local bone volume balance controls that may be associated with bone resorption and bone formation. Because bone resorption and formation both involve averaging over time, short-term fluctuations in the environment are removed, leaving the control systems to manage deviations in longer-term trends back towards their desired values. The length of time for averaging is much greater for bone formation than for bone resorption, which enables more filtering of variability in the bone formation environment. Remarkably, the duration for averaging of bone formation may also grow to control deviations in long-term trends of bone formation. Providing there is sufficient bone formation capacity by osteoblasts, this leads to an extraordinarily robust control mechanism that is independent of either osteoblast number or the cellular osteoid formation rate. A complex picture begins to emerge for the control of bone volume. Different control relationships may achieve the same objective, and the ‘integration of information’ occurring within a BMU may be interpreted as different sets of BMU control systems coming to the fore as different information is supplied to the BMU, which in turn leads to different observable BMU behaviors.

## Introduction

**Figure 1:**
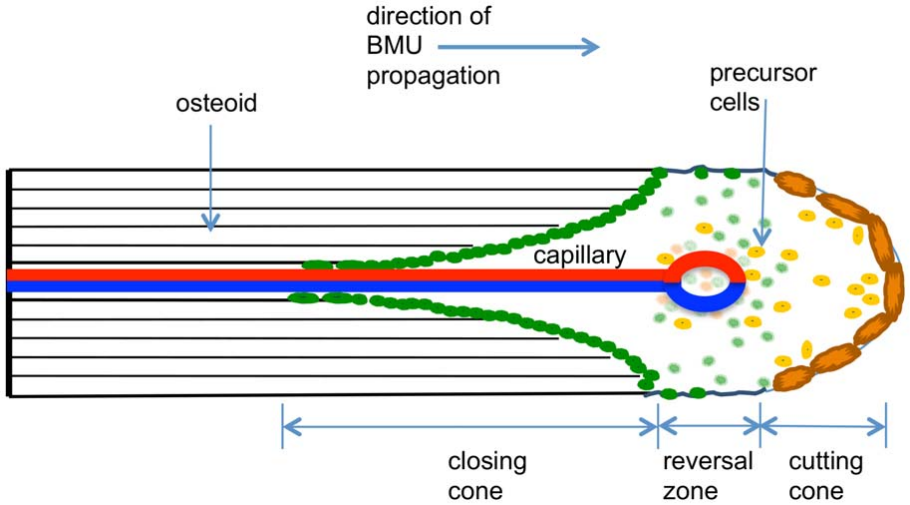
Idealized structure of cortical BMU in longitudinal section, showing cutting cone, reversal zone and closing cone. Cells of the osteoclast lineage are shown in light to dark orange while cells of the osteoblastic lineage are shown as light to dark green. A capillary is shown at the center of the BMU within a Haversian canal.

Organ tissues are comprised of groups of cells that coordinate their activities so as to achieve a functional outcome. In bone, the functional unit of cells is called a ‘basic multicellular unit’ (or BMU) [1]–[3]. BMUs are transient functional groupings of cells that progress through the bone, removing old bone and replacing it with new bone. A single BMU comprises active osteoclasts and active osteoblasts. Active osteoclasts resorb bone matrix at the front of the BMU, whereas active osteoblasts are found towards the rear of the BMU and form osteoid, which is later mineralized to form new bone matrix [4]. The cells within a BMU reside within the BMU cavity, which comprises the ‘cutting cone’, ‘reversal zone’ and ‘closing cone’ (see Figure 1) [5]. In a healthy adult, there are about one million BMUs operating at any one moment [6].

It has long been appreciated that bone formation is linked to bone resorption [7]. In bone physiology, ‘balance’ refers to a mode of BMU operation where the amount of bone resorbed equals the amount of bone formed [8]. Authors speak of the coupling between bone resorption and formation being ‘tight’, meaning that bone volume is held constant over long periods of time [9]. Precisely how an individual BMU is controlled to achieve and maintain bone balance has long fascinated researchers, as long-term imbalance may lead to clinically significant disease processes e.g. post-menopausal osteoporosis [10], [11]. Exactly how a BMU maintains homeostatic control of bone volume remains uncertain. Many correlations between variables have been established [9], [12]–[15] which are suggestive that they may be part of a larger ‘control system’, with many components operating simultaneously. The control of remodeling processes within a BMU is complex, and dealing with this complexity is a considerable obstacle to a deeper understanding of BMU operation, experimental investigation and to designing rational therapeutic interventions. The complexity of the control systems arises from the several sources.

One source of the BMU control complexity arises because a BMU integrates information gathered across multiple length scales [15], [16]. Information about the whole body is ‘fed’ into each BMU in the form of neuronal and hormonal signals through nerves and via blood vessels [15], [17]. At the length scale of the ‘whole body’, known signaling mechanisms include the sympathetic nervous system, the sex hormones (particularly the estrogens and androgens), corticosteroids (particularly the glucocorticoids), somatotropin (or growth hormone), thyroid hormones (including thyroxine and calcitonin), parathyroid hormone (PTH), vitamin D and its activated derivatives, and adiponectin, to name some of the better known systemic control molecules [13], [15], [17]–[21]. BMUs also receive information from a regional ‘tissue’ length scale. Over the last decade there has been growing evidence that osteocytes play an important role in interpreting conditions within the region of bone matrix immediately surrounding a BMU. Osteocytes feed this ‘regional information’ to the BMU and so modify its operation [22], [23]. The information is relayed via paracrine signaling molecules, including nitric oxide, prostaglandins, osteoprotegerin (OPG), the receptor activator of nuclear factor-κβ ligand (RANKL) and sclerostin, to name some the better known regional signaling molecules [23]–[26].

Within the BMU cavity itself, there are many additional intra-BMU signaling systems designed to maintain homeostasis. These include cell-cell contact signaling (e.g. notch [14], Eph-Ephrin [9], [27]) and membrane bound (and soluble) RANKL-RANK signaling [12], together with many autocrine and paracrine signaling systems [13]. There are many additional important regulatory molecules within the BMU cavity including proteases, inhibitors of proteases, binding proteins and extracellular matrix molecules including regulatory collagens, glycoaminoglycans, glycoproteins and various bone-specific molecules that have been identified, such as osteopontin and osteocalcin [18], [28]. We note that this list of signaling molecules is not exhaustive, and undoubtedly further signaling molecules will be discovered.

A further source of complexity arises from the inevitable interactions between all of the above-mentioned signaling systems. For example, the intra-BMU signaling molecules may ‘overlap’ with signaling molecules employed at different length scales, with molecules from both length scales influencing the operation of the BMU. The RANKL-RANK-OPG system operates at the whole body scale, the regional scale and within the BMU [25]. Further, within the BMU the type and magnitude of a signaling molecule’s influence on a particular BMU cell depends on the state of other signaling systems. This interaction dependence with other signaling systems is itself complex and non-linear, and can take many forms. For example, one signaling molecule may require another signaling molecule to be present for it to exert its biological effect. A case in point is the anabolic effect of PTH on osteoblasts, as the anabolic effect has been demonstrated to depend on a functional insulin-like growth factor (IGF) signaling system [13]. In this environment, identifying cause and effect is challenging, to say the least.

A further important source of control complexity arises from the fact that a BMU has multiple functional roles within bone. One known purpose of BMUs is to release minerals from the bone matrix, should this be required for blood mineral homeostasis [15]. For example, if calcium concentrations in the blood are too low, PTH is released from the parathyroid gland. A constant elevation of PTH stimulates an increase in BMU resorption of bone, which increases blood calcium concentration. A second purpose of the BMU is the repair of fatigue damaged bone [29]. Repeated loading induces the formation of micro-cracks in the bone matrix, which can grow, coalesce and may eventually result in a macro-sized fracture of a bone [30]. In this case the role of BMUs is thought to be to replace the old, damaged bone with new, undamaged bone, thereby improving the overall structural integrity of the bone [31]. Maintenance of the skeleton requires a net bone volume balance so that at the conclusion of the passage of a BMU, both bone mass and skeletal architecture are maintained.

Given that there are multiple purposes for a BMU, it is not difficult to imagine that the BMU may require a complex algorithm to ‘decide’ what to do in a given circumstance. In the parlance of control theory [32], a BMU requires multiple ‘objective functions’. It is also not difficult to imagine that the control systems within a BMU must in some sense ‘know’ which objective of the BMU takes precedence over another in a particular situation, and so some kind of priority needs to be established. For control this implies that the objective functions need to be weighted, perhaps in a time dependent way [15].

For example, hypocalcemia demands correction, as the consequences can be fatal for the organism. Hypocalcemia induces an increase in the secretion of PTH by the parathyroid gland, which then demands bone resorption. Under these circumstances, bone resorption to alleviate hypocalcemia may take precedence over maintaining bone volume required for structural integrity of the skeleton [15]. For an imbalance to occur, BMU controls need to be in some sense ‘overcome’. A reprioritization of BMU objectives presumably occurs, and the decreased risk associated with hypocalcemia is in effect traded for an increased risk of fracture associated with a decreased bone volume.

Finally, an important source of control complexity arises from having to deal with the inevitable variability within the environment. For any system to behave in regular and predictable way in the face of environmental variability, there are usually control systems in place to stabilize the system, such that it returns the system to its ‘set-point’ when perturbed. Otherwise small changes in the operating conditions may lead to wildly different system outcomes. Clearly an unstable BMU is incompatible with bone balance.

We have now identified many sources for the complexity associated with BMU control, and the challenge of understanding bone volume homeostasis is clearly daunting. The issue confronting us is how to make sense of available data on BMUs, and to turn this data into an integrated understanding of bone physiology that has explanatory power. This is typically the role of quantitative or theoretical models. There has been a fairly long history of mathematical and computational models of events in bone turnover. Earlier models, such as those summarized in Martin et al. [5] tended to focus on questions of rates of bone turnover e.g. what rate of resorption, mineralization, or BMU activation. More recently, computational models have been developed to model the evolution of various bone cell lineages and the role of specific signaling molecules [33]–[36]. Spatial aspects of cell organization within trabecular and cortical BMUs have very recently also been considered [37], [38]. These past models tend to be based on systems of differential equations.

A somewhat different approach to these past bone models is to look to ‘control theory’ [32], [39], [40] to provide some kind of framework for interpreting the available information on a BMU, as it deals with principles of control of dynamical systems. Indeed, that is what we will attempt to do in this paper and is the general conceptual approach that has been taken by others to understand bone regulation [15], [16], [35]. By doing this we hope to ‘step back’ from specific processes/interactions in a bone remodeling event and instead focus on the general requirements to achieve bone balance as well as the constraints this then imposes on various interactions. This should help to provide an explanation for observations in terms of control of bone balance and to systematically predict other currently unknown control mechanisms. In contrast, previous bone models of specific processes/interactions can be viewed as specific examples of these constraints known or assumed to occur.

The classical design control issue is ensuring a system maintains a constant single output for a single input. This is typically achieved using negative feedback control, so that the input is adjusted to achieve a desired output [40]. However, more complex systems with multiple processes also require control mechanisms to see that separate processes within the system are coordinated. Therefore within a BMU, we may expect to see negative feedback control(s) and process coordination control(s). Both mechanisms need to be considered for the homeostatic control of bone volume by a single BMU.

To make any progress, it is clear we first need to reduce the complexity described above. To do this, we would like the BMU to be operating in as simple a way as possible. So for the purposes of this study, we first assume that the BMU is established and steadily moving through the cortical bone (i.e. initiation and termination phases of BMU operation are not the focus of this paper). We further assume that signals from the ‘whole body’ level and from the ‘regional’ level to the BMU are in an averaged sense, time invariant. This enables us to focus on fundamental bone balance mechanisms operating within the BMU itself. This situation might be approximated in a young, healthy adult, with constant bone volume and normal bone turnover.

Even with these simplifications, there remain many signaling systems operating within the BMU, and no one is sure how the actions of these signaling molecules are integrated to maintain bone balance. To tackle this problem one may first ask: what needs to happen in a BMU to ensure bone balance is maintained? What are the different possible general ways that bone balance may be achieved? Having answered these questions one may then ask: what signaling processes and mechanisms within the BMU are potentially part of the BMU control systems to maintain bone balance? We will attempt tentative ‘answers’ to these questions. Of course for the reasons described above, the answers given here will necessarily be incomplete and obviously will need to be modified in the light of new information and new discoveries. Nevertheless, we hope the conceptual framework we employed here to analyze the BMU control problem will prove enduring.

In the following, we first describe a simple conceptual theory for maintaining constant behavior of any system. Then general control constraints are derived for maintaining bone balance. The closest analogous approach in biological systems is found within ‘Biochemical Systems Theory’ or ‘Metabolic Control Analysis’ [41]–[44]. However instead of focusing on just chemical populations (enzymes, metabolites etc) we will broaden our approach to include other state variables (e.g. cells, bone volume etc). We then focus only on a small sub-set of local controls that maintain bone resorption and formation. It will become apparent that there are many additional possible control constraints in a single BMU (see Table 1). Local controls operating for resorption, and for bone formation are then analyzed in detail. Finally, conclusions are drawn. Please note, a list of symbols and their definitions can be found in Table 2.

**Table 1:** Control relationships for bone balance within a BMU.

Control Rel. No.	Variables	Constants	Control Relationship
CR1	_k_^r^_, [OCa]_	_k_^f^_, [OBa]_	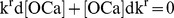
CR2	_k_^f^_, [OBa]_	_k_^r^_, [OCa]_	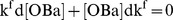
CR3	_k_^r^_, [OBa]_	_k_^f^_, [OCa]_	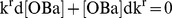
CR4	_k_^f^_, [OCa]_	_k_^r^_, [OBa]_	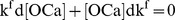
CR5	_k_^r^_, k_^f^	_[OCa], [OBa]_	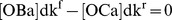
CR6	_[OCa], [OBa]_	_k_^r^_, k_^f^	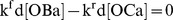
CR7	_k_^r^_, k_^f^_, [OCa]_	_[OBa]_	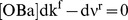 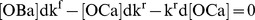
CR8	_k_^r^_, k_^f^_, [OBa]_	_[OCa]_	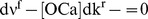 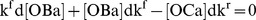
CR9	_k_^r^_, [OBa], [OCa]_	_k_^f^	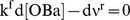 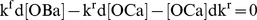
CR10	_k_^f^_, [OBa], [OCa]_	_k_^r^	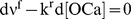 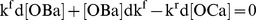
CR11	_k_^r^_, k_^f^_, [OBa], [OCa]_	__	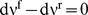 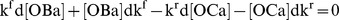

**Table 2:** Summary of symbols used and their definitions.

b^avOCa^	time average birth rate of active osteoclasts
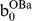	initial number of active osteoblasts at position z = Z of the closing cone
b^OBa^(τ)	birth rate of active osteoblasts at time τ
b^OCa^(τ)	birth rate of active osteoclasts at time τ
G	function relating model state variables to model outputs of interest
G_k_	k^th^ system output of interest
G_k0_	equilibrium or reference state of the k^th^ system output of interest
_k_^f^	average rate of bone (or osteoid) volume formed by each cell
_k_^r^	average rate of bone volume resorbed by each cell
_L_^cc^	length of closing cone
M	λ^OBa^ + λ^kf^
[OBa]	active osteoblast number
[OCa]	active osteoclast number
_r_	radius of the closing cone
_rc_	Haversian canal radius required for bone balance
r^th^	thickness of bone at z = Z measured in the radial direction
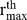	upper limit on the amount (radial thickness) of bone that can be formed
r_0_	resorption cavity radius at some location z = Z along the closing cone
r_1_	current Haversian canal radius at some location z = Z along the closing cone
_s_^*OBa^	assumed specific form of the active osteoblast survival curve s^OBa^(t − τ)
s^OBa^(t − τ)	active osteoblast survival curve
s^OCa^(t − τ)	active osteoclast survival curve
_t_	time
_T_^avOCa^	average lifespan of active osteoclasts
T^f^	time required for the bone at a cross-section of the closing cone to achieve the radius of the Haversian canal
ν^BMU^	average velocity of the BMU
ν^f^	total rate of bone formation by all active osteoblasts in the BMU
ν^rcc^	total volume of bone formed by osteoblasts at some time t over the length of the closing cone
ν^r^	total rate of bone resorption by all active osteoclasts in the BMU
_z_	position along long axis of the cutting cone with z = 0 at point of cone closure
_Z_	specific position along z-axis of closing cone
*_Greek_*	
α_i_	i^th^ model state variable
α_i,0_	equilibrium or reference value of the i^th^ model state variable
λ^kf^	decay constant used in the bone formation rate per cell, Equation (22)
_λ_^OBa^	decay constant used in the active osteoblast survival curve, Equation (21)
_χik_	system sensitivity of the k^th^ model output to the i^th^ model state variable, as defined by Equation (2)

## Methods

We first seek a general conceptual framework that will allow us to interpret existing data, make predictions, and so better understand how controls within a BMU may be arranged to maintain bone balance.

Let the desired output of a system (e.g. bone volume) be called the reference output. Let the desired reference output be the result of reference inputs (e.g. cell numbers, rate of bone formation per cell or rate of bone resorption per cell). The inputs are referred to as ‘state variables’ of the process. In essence, a controller typically manipulates the state variables so that the process delivers, as closely as possible, the desired reference output (in our case, constant bone volume) [32], [40]. Typically, a controller compares the actual input with the reference input, and changes the input to minimize any difference. This is called negative feedback control and is essential for homeostasis.

Negative feedback control is typically implemented using proportional, integral or derivative controllers or some combination of the three [40]. For biological systems, the information needed for feedback control is often carried via soluble chemicals or on the surface of cells interacting in a region of space. A negative feedback proportional controller may involve a signaling molecule interacting with a cell, and the cell responding by producing a ‘decoy’ molecule, which in turn limits the strength of the original signal. The amount of signal attenuation depends ‘proportionally’ on the strength of the signal, though in most cases the controller includes non-linearities, such as saturation [45]. However, proportional feedback control of an input cannot return the input to exactly the reference value, as doing so removes the drive to the controller [40]. Therefore, proportional control usually delivers a residual deviation from the desired reference output. If one desires the feedback controller to return the output variable to exactly the desired reference value, then a negative feedback integral controller is usually employed. Finally, if one wants a controller that anticipates future states, then a derivative controller may be employed. This type of control could be part of a predictive controller. We will try to identify proportional, integral and differential control mechanisms for maintaining bone volume homeostasis.

Let some model of a process (represented by a function G) relate the model state variables to model outputs of interest. Let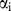 represent the i^th^ model state variable and G_k_ is the k^th^ system output of interest. For small changes in state variables about an equilibrium state 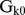, it may be found (using the first two terms of a Taylor series) that,
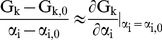
(1)System ‘sensitivity’ (χ_ik_) [46] can be defined, which compares the change in a system output to a change in each of the model state variables, viz,
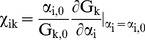
(2)If the state variables are independent, a new value of G_k_ can be predicted from an old value G_k,0_ using the sensitivity parameters, viz,
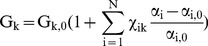
(3)For homeostasis of some process represented by the model (e.g. maintaining bone volume), we require G_k_ = G_k,0_. For this to happen, Equation (3) requires,
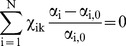
(4)This equation gives a constraint condition describing how increments in the state variables 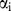 need to be related to one another so as to achieve constant output. Note that a constant system output is required for homeostasis.

Using this general conceptual framework, we can identify possible control constraint equations that a single BMU may employ for bone balance. Our methodology is to start with a bone volume ‘conservation equation’, and then introduce ‘constitutive relationships’ that involve the state variables. A wide variety of constitutive relationships may be chosen, but here we err towards simple constitutive relationships. We then identify control constraint equations (or control relationships) among state variables describing bone volume, so that Equation (4) is satisfied. In a few cases, we illustrate the control relationships that may be implemented within a BMU based on reported experimental research findings. However it is important to note that to date there are no reported experiments investigating the operation of a single BMU.

The reason we adopt this methodology is that we believe the control mechanisms for maintaining bone volume balance are likely to be constructed from the control constraints derived here. Acting in concert, a set of process control equations identified here will enable a BMU to maintain constant bone volume in the face of variability. We now seek a suitable set of control constraint equations for changes of bone volume.

Our focus is on a single BMU. In the case of bone turnover in a young, healthy adult, we first require that bone formation by the BMU be exactly equal to the bone resorption by the BMU. That is,
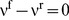
(5)where ν^f^ is the total rate of bone formation by all active osteoblasts in the BMU, and ν^r^ is the total rate of bone resorption by all active osteoclasts in the BMU. In other words bone volume remains constant. For balance to occur, we note that the rates of bone formation or resorption may change over time and so for example bone turnover may increase, but any change that occurs in bone formation and resorption needs to be equal, so that,
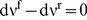
(6)where dν^f^ is an increment in the total rate of bone formed by all active osteoblasts within the BMU, and dν^r^ is an increment in the total rate of bone resorption by all active osteoclasts within the BMU. For bone volume balance we require that Equation (5) and Equation (6) be satisfied. If this occurs then balance is maintained, that is bone volume remains constant even when bone turnover changes.

We now introduce ‘constitutive equations’ describing how the increments in the rate of bone volume formed may be achieved. We first assume a very simple constitutive equation, namely, that the bone formed in a given period of time is equal to the product of the active osteoblast number ([Oba]) and the average rate of bone (or osteoid) volume formed by each cell (k^f^), viz,
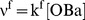
(7)


In this case, an increment in the rate of bone volume formed is given by,
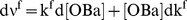
(8)


A similar constitutive relationship may be employed for the rate of bone resorption by active osteoclasts ([OCa]), and so an increment in the rate of bone volume resorbed is given by,
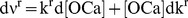
(9)Now substituting Equations (8) and (9) in Equation (6) leads to,
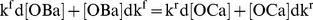
(10)This is our general control constraint equation. This is a very general statement of the relationships between increments of the state variables (the state variables being k^f^, k^r^, [OBa], [OCa]) that are required to ensure that balance is maintained over time.

Equation (10) actually implies a ‘family’ of potential control relationships for maintaining bone balance under various conditions. For example, if all incremental variables are nonzero then Equation (10) describes the relationship between them. Alternatively if any one of the four incremental variables is set to zero, then there are four combinations of relationships between the remaining variables. Likewise, assuming any two of the incremental variables are set to zero, then there are six combinations of relationships between the remaining variables. This gives a total of eleven different control relationships.

Each of the eleven control equations that may be employed to deliver constant bone volume in a single BMU is shown in Table 1. However, while one would expect that some of these control relationships are implemented in a BMU, it is not necessary (or even desirable) for all of them to be implemented. Indeed, some might be contravened if there is a more important BMU objective than bone volume homeostasis. For these reasons, the eleven control relationships should be considered as a guide to developing potential control systems, some of which may be implemented within the BMU, and some of which may not.

## Results and Discussion

### Investigation of Control Relationships within a BMU

As it is not possible to examine all the implications of the control equations shown in Table 1, in this paper we only focus our attention on the first two of the control equations, that is, equations CR1 and CR2 (shown in Table 1). For each control relationship, we first discuss what each of these relationships implies and then consider how each relationship describing bone resorption and bone formation may be implemented within a BMU. We then look at experimental research reports in the literature that might suggest such a control relationship occurs within the BMU.

While often there is much that is suggestive and thought provoking, unfortunately many biological research experiments involve ill-defined systems, or use very different physical models under a huge variety of environmental and initial conditions, and often report a very limited set of observations on the system. For example, experiments may take place in different species, or use different cell cultures, often in a variety of ill-defined conditions. Alternatively, ‘conditioned media’ is often added to cell cultures, but the composition of the conditioned media is not defined or its relevance to in vivo conditions is unclear. In addition, many experiments take place under conditions far from normal (e.g. gene knockout studies). Further, many experiments have not yet been performed, and undoubtedly much remains to be discovered about BMUs (there are for example, no reports of experiments that directly interrogate a single isolated BMU).

For all these reasons, interpreting research findings in terms of BMU bone volume homeostasis needs to be done cautiously, with any implications for bone balance being tentative until verified. Of course confronted with such uncertainty our interpretations are necessarily approximate and incomplete, and so our conclusions are necessarily tentative. With this in mind and with appropriate caution, let us now see how the control relationships one and two in Table 1 may provide us with a framework to help us understand how a BMU maintains bone balance.

### Control Relationship 1: CR1: 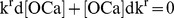


Bearing in mind the assumption of constant bone formation rate, we then seek ways to maintain bone balance when state variables change. Equation CR1 implies that there needs to be an inverse relationship between the osteoclast number and individual osteoclast resorption rate. That is, as the number of osteoclasts increases the rate of resorption per osteoclast decreases, or vice versa. Note the relationship only involves one cell type, active osteoclasts, which are located within the cutting cone at the front of the BMU. As such it is likely that this control relationship is ‘local’. This knowledge helps to focus our attention on ‘signaling systems’ located within the front of the BMU.

Now the number of osteoclasts is by definition given by,
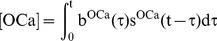
(11)where b^OCa^(τ) is the birth rate of osteoclasts at time τ and s^OCa^(t-τ) is the osteoclast survival curve. This ‘convolution integral’ simply acknowledges that the present number of osteoclasts is a function of the time history of osteoclast production and osteoclast survival. It is apparent that the osteoclast number at any one time is a time-weighted average of prior osteoclast production rates and apoptosis rates. Averaging acts like a (‘low-pass’) filter, smoothing out ‘short-term fluctuations’ in the environment, leaving any longer-term trend. If we define the duration of the time averaging, we can then define what we mean by short-term fluctuations, and what we mean by longer-term fluctuations that will produce a trend.

As the lifespan of osteoclasts in humans is about ten days, an appropriate averaging period is about ten to fourteen days [6]. This means fluctuations on the time scale of less than a day or so would be considered short-term (e.g. daily fluctuations in hormone levels influencing the osteoclast production rate). Short-term fluctuations do not require active control relationships, as they are simply ‘averaged out’ by the time integration shown in Equation (11). Averaging over time is an important mechanism for increasing stability of the system. However, environmental fluctuations over a period of several days or weeks may well produce a trend in osteoclast numbers (e.g. regions of higher growth factor concentration in the bone matrix may lead to sustained increases in the osteoclast production rate, or vice versa), and so fluctuations on this longer time scale would need to be controlled through active control processes if bone resorption is to be maintained at a constant rate. Control systems in effect ‘manage’ the trends back towards a desired normal.

Let us assume first there are no control processes in operation and that b^OCa^ and s^OCa^ are independent variables. In this case Equation (11) is equal to,
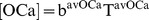
(12)where b^avOCa^ is the time average birth rate of osteoclasts and T^avOCa^ is the time average lifespan of osteoclasts. Inserting Equation (12) into our control relationship CR1 leads to,



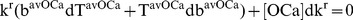
(13)To relate this specific equation to the previous discussion and general equation (Equation (4)), we note that the ‘alphas’ (α_i_) for this equation are T^avOCa^, b^avOCa^, and k^r^, while the sensitivity coefficient (χ) for dT^avOCa^ is k^r^b^avOCa^, for db^avOCa^ is T^avOCa^ and finally for dk^r^ is [OCa].

While it is possible to consider Equation (13) directly, to help appreciate the significance of Equation (13) more fully, let the average birth rate of osteoclasts be held constant. In this case, Equation (13) simplifies to
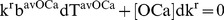
(14)This equation now suggests there may be an inverse relationship between the rate of resorption and the average lifespan of an osteoclast. Bearing in mind the assumptions, Equation (14) furthermore quantifies the ratio of the magnitude of incremental changes in osteoclast number and the average lifespan in terms of measurable variables (actually the ratio of sensitivity coefficients) required to maintain a constant osteoclast number, viz,



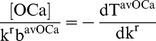
(15)Though rather extreme conditions (which may be required to enable the relationship to be clearly observed using the experimental methods currently available), such an inverse relationship has been described in the clinical literature. Karsdal et al. [47] reports:

Using inhibitors of both ClC-7 and the V-ATPase we have provided evidence that inhibition of acidification prolongs osteoclast life span.

So in this case a mechanism enabling the inverse relationship between variables in Equation (14) is suggested in the literature, namely, that a decrease in the rate of resorption by osteoclasts decreases their rate of apoptosis, and so increases their lifespan. This inverse relationship between rate of resorption and average osteoclast lifespan is consistent with an osteoclast resorbing bone matrix until the resorption pit beneath the osteoclast reaches a pre-determined average depth, and the osteoclast then undergoes apoptosis [6], [47], [48].

Next, let the average lifespan of osteoclasts be held constant and allow the active osteoclast average birth rate and the rate of resorption to vary. In this case Equation (13) simplifies to,
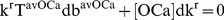
(16)This equation suggests there may be an inverse relationship in the BMU between the rate of resorption and the osteoclast birth rate. A report of such an inverse relationship is difficult to find in the literature, however. This does not mean such a relationship does not exist. A possible explanation is that it does in fact exist but that it is not prominent enough to have been detected using current experimental methodologies. Let us continue with our examination of Equation (13) before considering Equation (16) again.

Now assume that the average rate of resorption by individual osteoclasts is held constant, while the active osteoclast average birth rate and life span vary. In this case Equation (13) simplifies to,
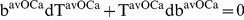
(17)This equation suggests there may be an inverse relationship in the BMU between the average lifespan of the osteoclasts and the average osteoclast birth rate. That is, if the rate of apoptosis of osteoclasts is increased then their rate of replenishment needs to increase so that osteoclast numbers are maintained. Maintaining osteoclast numbers is actually a fundamental requirement for BMU operation to be sustainable. Given that BMUs in long bones exist for periods of months, compared to a lifespan of weeks for osteoclasts [2], [3], [6], [23], we might expect that there would be several feedback mechanisms to ensure a relationship between the supply of new osteoclasts and their rate of removal in the cutting cone. One general mechanism suggested in the literature is that factors released from the bone matrix stimulate osteoclast development [6].

If we acknowledge that matching the supply of osteoclasts to their rate of removal is an essential control requirement for the existence of a BMU, Equation (17) may take precedence over control requirements of lesser importance, such as a control requirement for bone balance. Upon reflection about prioritization of objectives, this is perhaps to be expected. However this realization may help explain why Equation (16) is at least not prominent, for if Equations (14) and (17) are operational as described above, then Equation (16) potentially conflicts with Equations (14) and (17). To explain this – Equation (14) requires that an increase in resorption rate decreases the lifespan of the osteoclast, and as a consequence, Equation (17) and the decrease in osteoclast lifespan requires there be an increase in the birthrate of osteoclasts. But if this pair of relationships is operational as postulated above, this is incompatible with Equation (16), which requires that an increase in the resorption rate cause a decrease in the birth rate of osteoclasts.

So while Equation (16) could in principle provide a useful bone balance control, it may not be implemented because it conflicts with the sustainability of the BMU. Indeed, this conflict suggests that if the BMU objective were not bone balance, but the BMU objective was say cessation of its own operation, then Equation (16) may be a useful part of a control system to shut down BMU operation.

We may notice that if Equations (14) and (17) are operational as described above and resorption increases, then together they imply that the total bone volume resorbed per unit time will increase. As noted in the discussion of Equation (6), this is not incompatible with bone balance (it requires that the rate of bone formation be increased to match the new rate of removal). Increased bone resorption per unit time, may be useful for changing the diameter of the resorption cavity while maintaining constant BMU speed, or it may be useful for changing both BMU speed and resorption cavity diameter.

Interestingly, this observation about Equations (14) and (17) and BMU speed leads us to reconsider Equation (16). For if we now have the objective of controlling BMU speed, then Equation (16) may be a useful part of a control system. Indeed, we see that a control system managing the speed of the BMU is not incompatible with a control system managing BMU shutdown (as zero speed is necessary for BMU shutdown and BMU shut down may be regarded as an extreme control state for BMU speed).

This brings us full circle. For if we have the objective of controlling resorption, then Equation (16) may be a useful part of our control system, for as the BMU speed is reduced Equations (14) and (17) no longer imply osteoclast numbers increase. With Equation (15) operational alongside Equations (14) and (17), the volume of bone resorbed may be held constant. Indeed, we are now considering varying all three variables in Equation (12) simultaneously (which was its original form before we simplified it to three, two variable equations).

We now see that our analysis of Equation (12) has taken us on a short but illuminating journey and enabled us to see how bone balance can be maintained by two competing processes (one driving the BMU faster, the other slowing the BMU down). Because bone balance may be composed of two or more processes ‘pulling’ in different directions, we begin to uncover how the objective of balance may be transformed into other BMU objectives – such as that the BMU exist, that the BMU may change its speed of progression or that a BMU may cease its operations. All of these BMU objectives may be realized when different emphasis is given to the control relationships for bone balance. However taken together in the right proportions, they are also compatible with the general objective of bone balance.

Indeed the control system defined by Equation (13) (or even more generally, by Equation (10)) is actually in operation. These complete control equations potentially allow all variables to vary simultaneously, but in a coordinated way so that balance is maintained. However the discussion above, stimulated by holding one of the three variables constant at a time, is helpful when attempting to discover the underlying processes enabling Equation (13) to be satisfied, or when considering the possible dominance of these various processes, recognizing that a BMU may have a need to reprioritize objectives.

From the discussion above, we see a complex picture emerging for control of bone volume. We can see that different control relationships may achieve the same end, but when some additional requirements are placed on the system, some control relationships for a particular objective may no longer be viable. And we see that control relationships that were not viable for one objective may become useful for other objectives. Indeed, the ‘integration of information’ within a BMU may be interpreted as different sets of control systems coming to the fore as different information is supplied to the BMU, which in turn may be interpreted as different BMU objectives, inferred from associated observed BMU behaviors.

### Control Relationship 2: CR2: 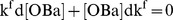


We now turn to control relationship 2 in Table 1. Like CR1, Equation CR2 implies an inverse relationship between the active osteoblast number and the active osteoblast formation rate. That is, as the average number of osteoblasts increases, the average rate of bone formation per cell decreases, and vice versa. Again it is most probable that control relationship CR2 is also ‘local’, because it involves only one cell type, active osteoblasts, which are located within the closing cone towards the rear of the BMU. This knowledge helps to focus our attention on signaling systems located within this region of the BMU. However osteoclasts are relatively long lived, and so to better understand what CR2 implies, we need to consider what is happening along the length of the whole closing cone.

Importantly, it is believed osteoblasts do not move very far from their origination point, generally staying at the location where the developing osteoblasts first attach to the wall of the nascent osteon (towards the rear of the reversal zone). The cells of the lineage of developing osteoblasts extend from the region around the tip of the BMU blood vessel to the wall of the reversal zone, a journey that probably averages about two and half to three days [49]. On contact with the reversal zone, developing osteoblasts differentiate into active osteoblasts and begin osteoid formation [5]. Osteoblasts then begin to fill the resorption cavity with osteoid, which later mineralizes to bone (Figure 1). As the osteoid fills the closing cone cavity, the radius r of the closing cone reduces over time, from the initial resorption cavity radius (i.e. the future osteon radius) to the final radius of the Haversian canal (for simplicity, we consider the BMU to be rotationally symmetric).

Because active osteoblasts do not move very far in the longitudinal direction along the closing cone, it is interesting to contemplate the fact that as one moves along the closing cone towards the rear of the BMU, it is like ‘looking’ further and further back in time. The closing cone becomes a faithful record of past events, and this record can be utilized for dating purposes and inferring rates of bone formation [5].

The closing cone length can be measured in millimeters, indicating that osteoblasts may survive many months (the average lifespan in humans is quoted as averaging 150 days [4], [5]). Because there is a correlation between time and distance along the closing cone, depending on what best suits our purpose, we may use distance and time interchangeably to identify locations along the closing cone. As a result of this interchangeability of distance and time, we can count active osteoblast numbers by integrating over the length of the closing cone, or equivalently we can take an integral over time (i.e. as for Equation (11)). We begin by writing the total number of osteoblasts both ways, and choose one or other approach depending on our purpose. We take the origin of the z-axis to be at the point of cutting cone closure, which coincides with time zero.

Then the number of osteoblasts in a BMU observed at some time t is given by,
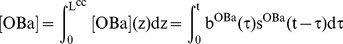
(18)where L^cc^ is the length of the closing cone, [OBa](z) is the number of active osteoblasts at each location z along the closing cone at time t, b^OBa^(τ) is the birth rate of active osteoblasts (production rate defined as the total number of new active osteoblasts per time) at time τ and s^OBa^(t-τ) is the osteoblast survival curve (reflected in time).

We note in passing that because the birth rate of osteoblasts (b^OBa^(τ)) is a process lasting on average two and half to three days [49], then the birth rate of active osteoblasts is itself a time-averaged function of this formation process. Short-term fluctuations in osteoblast formation, perhaps up to a day or so, are filtered out during the osteoblast formation process. It is possible that the time period of fluctuations that are effectively filtered out may become shorter as the diameter of the resorption cavity decreases (assuming that the time spent in the proliferative stage of the osteoblast formation process also decreases). This may lead to an increased variability in b^OBa^(τ) as the initial resorption cavity diameter decreases.

Now by including the rates of bone formation per cell in the integrations, we can also calculate the total volume of bone formed by osteoblasts at some time t over the length of the closing cone, viz,
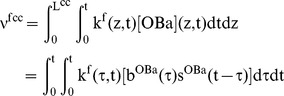
(19)


Given the bone volume formed is to be matched with that resorbed, we only require that the final volume of bone along a closing cone is equal to that resorbed. In theory, the radius of the Haversian canal could vary unevenly along its length and still be consistent with bone balance. This undoubtedly happens to varying extents in vivo [50]. However for convenience, here we assume that the internal radius of the Haversian canal is constant.

Now because each cross-section of bone may be considered independent of nearby cross-sections, we can focus on a particular cross-section (say denoted by location Z), and consider some representative interval centered at z = Z, perhaps up to few osteoblasts in width (the interval size could be varied to approximate the progression of a BMU in about one day). We can then consider our control relationship (CR2) for each cross-section of bone. CR2 requires that at each cross-section, a differential increment of bone formed at that cross-section must be zero, viz,
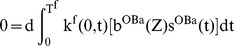
(20)where T^f^ is the time required for the bone at a cross-section to achieve the radius of the Haversian canal. To be clear, we note that b^OBa^(Z) is constant at each location Z, but b^OBa^(z) may vary with distance along the closing cone. Because b^OBa^(Z) represents the initial number of OBas at a particular location Z, we later denote it by 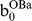 in equations below.

Very importantly, we note that (i) T^f^ is measured in months, and that (ii) T^f^ is variable. The variability of T^f^ is evident visually as the length of the closing cone is variable. Based on our previous discussion of filtering, we may expect that because there is an integral over time that is measured in many months, we might expect that the time averaging will be an effective filter for longer period fluctuations. This is true. Because the length of time for averaging is so long – ‘short-term’ fluctuations may now be measured in several weeks (as opposed to days used for CR1). This integral over time effectively averages out the environmental fluctuations, including that variability associated with changes in osteoblast number or with their cellular rate of osteoid formation or both.

However this averaging mechanism is also a control mechanism, because trends in the bone formation rate over time are also brought back towards normal by varying the time interval for bone formation. One could view this control mechanism as being a ‘variable filter’ that dynamically adjusts itself to filter out fluctuations in rates of bone formation. When the time period of the fluctuations in bone formation increases, then the length of the time integration also increases.

The physical mechanism for varying the filter properties is by changing the closing cone geometry – the surface area available for active osteoblasts to form bone changes dynamically with the bone formation rate. If the bone formation rate is low, the length of the closing cone and its surface area increases, and vice versa (this is evident because integrals of time t may be replaced by integrals over distance z, so that if T^f^ increases then so does the length of the closing cone). An increase in surface area enables active osteoblast numbers to increase, and vice versa.

To gain more quantitative insight into the process of dynamic adjustment of the closing cone, we first consider a cross-section of bone, and introduce two constitutive equations. The constitutive relationships introduce new parameters describing osteoblast survival and cellular bone formation rate, which reflect the tissue specific circumstances observed during bone formation.

We assume that the active osteoblast survival curve is an exponentially decreasing function with time, viz,
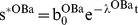
(21)and that the bone formation rate per cell is an exponentially decreasing function with time, viz,



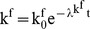
(22)Using these constitutive equations, the thickness of bone formed in a radial direction at each section may be calculated. Let r_0_ be the resorption cavity radius, and r_1_ the current Haversian canal radius at some location z = Z along the closing cone, then,
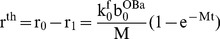
(23)where 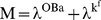
and r^th^ is the thickness of bone at z = Z measured in the radial direction. If 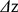
is the width of the cross-section, then the volume of bone at each cross-section (as a function of time) is given by,



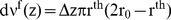
(24)These are plausible forms for the constitutive equations because they lead to results that are consistent with the quantitative results of tetracycline labeling studies [51]–[53]. To demonstrate this, we simply take the differential increment of bone radius with respect to time to obtain,
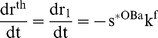
(25)That is Equation (25) is consistent with reported data [51]–[53]. This indicates that bone formation rates decrease exponentially along the closing cone.

We immediately see from Equation (23) that there is an upper limit on the amount of bone that can be formed, and that there is a radial thickness of bone that can never be exceeded (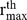). That is,
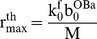
(26)Clearly, if 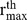 is less than 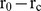, where r_c_ represents the Haversian canal radius required for balance, then bone volume balance is simply not possible.

Equation (23) also indicates that if the bone formation capacity is low, then M needs to decrease in order to maintain a constant r^th^. When r^th^ is greater than 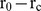 and r^th^ decreases for any reason, more time is required to form the amount of bone required for balance. This extension in the time for bone formation is actually realized by increasing the length of the closing cone, and represents a fundamentally important mechanism for maintaining balance in bone. The ‘beauty’ of this physical mechanism is that it automatically adjusts itself to enable bone volume balance. The mechanism only fails to match bone formation with bone resorption when the maximum bone formation potential of the cells is less than the bone volume resorbed (i.e. it only fails when 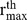
is less then 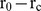).

It is also interesting to contemplate the maximum osteoid formation capacity relative to that required. In other words, what ‘factor of safety’ may be on the ‘total osteoid production capacity’ of osteoblasts, and how does this factor of safety evolve with age? It is likely that the factor of safety on osteoid production capacity of osteoblasts is a function of many factors, including system, regional and locally produced signals that sustain the osteoblast population and their bone formation capacity within the BMU.

Of course even if balance is theoretically possible, balance ‘failures’ of a practical nature may occur. For example, if the formation rate is too slow there may be insufficient time available to restore the bone volume. One can imagine other kinds of failures occurring. For example, the increased size of the BMU cavity as a result of the increasing closing cone length may compromise the overall structural integrity of the bone. It is desirable to minimize the size of BMU cavity so as to maximize bone volume, thereby decreasing the risk of bone fracture. Clearly there is a risk and cost associated with decreased bone formation rates, even when bone volume is maintained.

Because of the interchangeability of t and z, Equation (23) also describes the shape of the closing cone in longitudinal section (i.e. radial thickness of bone with distance along the closing cone). If the initial number of OBas (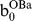) is constant along the length of the closing cone and the velocity of the BMU is also constant, then the closing cone shape assumes a particularly simple form, viz,
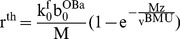
(27)where v^BMU^ is the average velocity of the BMU (for this equation, z is measured from the initiation of the closing cone). We may also take the differential change of the radial bone thickness (i.e. Equation (24)) and setting the result to zero, we obtain an expanded version of control relationship CR2 at a particular cross-section of bone, viz,



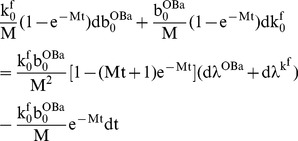
(28)Again we may set various combinations of parameters to zero, and so simplify the system so as to facilitate understanding. However, we note that there are now five separate state variables, and if these are taken in combinations two at a time, there are ten possible sets of constraints. For the sake of brevity, we only consider two of the possible combinations.

We first set dt in Equation (28) to zero. The resulting control equation suggests that an increase in the bone formation rate needs to be matched by an increase in the M. The character of this control equation (i.e. dt in Equation (28) set to zero) is actually the mechanism required during normal bone formation processes within a rotationally symmetric BMU, for as osteoid is secreted by the osteoblasts, the surface area available for cell contact continually decreases.

Effectively, the secretion of osteoid by active osteoblasts leaves the osteoblasts with less space, and less contact area for osteoblasts to interact with osteoid. The continual reduction in contact area and space sustains ongoing competition between the active osteoblasts for contact area, space and nutrients. Eventually some osteoblasts are forced off the contact surface. Without contact with osteoid and pro-survival signaling through adhesion molecules the active osteoblasts undergo apoptosis [54], [55]. On the other hand, osteoblasts that are most successful in the competition for contact with the osteoid are more likely to undergo a transformation and terminally differentiate into osteocytes. Clearly high rates of osteoid formation (i.e. from the presence of many osteoblasts and high bone formation rates) leads automatically to high rates of apoptosis, and high rates of bone formation probably lead to increased numbers of osteocytes. Both of these processes decrease osteoblast survival, that is, when osteoid formation rates are high both processes decrease osteoblast number at some future time. The mechanisms responsible for this inverse relationship are exactly the same ones implied by Equation (28) with dt set to zero. These fundamental control processes [54], [55] for bone formation are consistent with control relationship two (i.e. CR2) in Table 1.

The special feature of Equation (28) is that time is also present as a variable, and T^f^ can be increased as large as desired so as to ensure that the final bone volume is maintained constant at each cross-section of bone (dt in Equation (28) increases as T^f^ increases). As noted previously, this is contingent upon active osteoblasts actually having a capacity to remain alive and continuing to produce osteoid. If they do not, then the bone volume formation falls short of that required for coupling, even when time increases without bound.

We have seen that the closing cone is a remarkable physical device that both smoothes variability in the environment and acts as an integral controller of the BMU bone formation rate, ensuring coupling is maintained. Critically important is the observation that the number of preosteoblasts produced does not exactly need to match some predetermined number of active osteoblasts, as the number of osteoblasts at a cross-section of the closing cone is not fixed. Preosteoblast production rates can be variable, and initial numbers of active osteoblasts can also be variable, yet balance in bone volume will still occur. Indeed, the capacity of the closing cone to change its surface area and so support a greater number of active osteoblasts when their formation rate is lower, means there is no unique cellular rate of production of osteoid required to ensure balance. The only critical requirement for balance is that the total production capacity of the osteoblast population is sufficient to replace the bone removed.

Based on the foregoing discussion, one would expect that when the variability in the environment increases, so does the standard deviation in the length of the closing cones, and when the maximum rate for osteoblasts to form bone is reduced, the average length of the closing cone increases. In other words, the variability in the length of the closing cone is providing information on the variability of the BMU environment, while changes in average length of the closing cone in BMU provides important information on osteoblast capacity to produce osteoid. Simply stated, short closing cones indicate high capacity to form bone, while long closing cones indicate low capacity to form bone.

### Conclusions

We have established a qualitative and quantitative framework for analyzing bone volume balance control in a single cortical BMU. This theoretical framework offers the possibility of a change from the near dominance of discovery led bone research, towards a directed search for processes compatible with the control constraint equations that arise from this theoretical framework. The essence of the approach is to express the differential change in bone volume as the difference between differential changes in bone formation and resorption, and then introduce suitable constitutive equations. By setting some state variables to zero, a family of differential equations is found that may be applied to both better understand and to identify candidate processes involved in maintaining bone volume in bone following transit of a single BMU. After deriving a general family of control constraint equations, we focus our attention on just two control constraint equations suitable for the understanding and identifying local controls that may be acting during bone resorption or bone formation.

It is found that averaging enables short-term fluctuations in the environment to be smoothed out, so that control processes need only be focused on long-term trend deviations. Substituting constitutive equations of any desired complexity into the differential control constraint equations enables insights into relationships between the system state variables required for homeostasis. Simple constitutive equations are employed here. We find some experimental evidence for the theoretically postulated inverse relationship between rate of resorption and average osteoclast lifespan. We also find that bone balance may be maintained by two competing processes (one driving the BMU progression faster, the other slowing the BMU down). Because the processes leading to bone balance may be composed of two or more processes each ‘pulling’ in different directions, we begin to uncover how the objective of maintaining bone may be transformed into other BMU objectives – such as that the BMU exist, that the BMU may change its speed of progression, that bone turnover occurs or that a BMU may cease its operations. All of these BMU objectives may be realized when different emphasis is given to the control relationships for balance. However if these control relationships are taken together in the right proportions, they are also compatible with the objective of bone volume balance.

Bone formation takes place in the closing cone, which may vary in length. Actively secreting osteoblasts do not move very far from their original point of attachment. The peculiarities of the bone formation process result in a remarkable variable averaging process that enables both short and long-term fluctuations in the environment to be ‘averaged out’. This is, in effect, a powerful integral controller that acts independently to match bone resorption with the objective of bone balance, without the need for any formal communication between the two processes of bone resorption and formation (though formal communication systems may play a role too). The only critical requirement for balance is that the total production capacity of the osteoblast population is sufficient to replace the bone removed. Quantitative analysis of the closing cone dynamics reveals they are consistent with the theoretical control prediction that osteoid secretion per cell varies inversely with osteoblast number. Theoretical equations have been derived for control relationships within the closing cone, and the closing cone’s shape and length.

Of course, many signaling molecules are known to provide additional information to assist in coupling bone resorption and formation. These processes include the release of growth factors from the bone matrix during resorption (and from the osteoclasts themselves), and the release of OPG and soluble RANKL by cells of osteoblastic lineage. Such signaling processes can be analyzed using the remaining differential equations in the family of control constraint equations shown in Table 1. These analyses will be the subject of future work.
